# Nucleolar Cdc14 Splitting Reflects Recombination Context and Meiotic Chromosome Dynamics

**DOI:** 10.3390/ijms27020888

**Published:** 2026-01-15

**Authors:** Patricia Rodríguez-Jiménez, Paula Alonso-Ramos, Isabel Acosta, David Álvarez-Melo, Jesús A. Carballo

**Affiliations:** 1 Institute of Functional Biology and Genomics (IBFG), Spanish National Research Council (CSIC-University of Salamanca), 37007 Salamanca, Spain; 2 Centro de Biotecnología y Genómica de Plantas, CBGP, CSIC-INIA, Campus de Montegancedo, 28223 Madrid, Spain; 3 Louvain Institute of Biomolecular Science and Technology, Université Catholique de Louvain, 1348 Ottignies-Louvain-La-Neuve, Belgium

**Keywords:** biomolecular condensates, Cdc14 phosphatase, meiosis, meiotic recombination checkpoint, nucleolus, *Saccharomyces cerevisiae*

## Abstract

Chromosome dynamics, recombination, and nucleolar organization intersect during meiotic prophase I, yet how the recombination context influences nucleolar architecture remains unclear. We analyzed the nucleolar pool of Cdc14 in *Saccharomyces cerevisiae* under matched prophase I gating and a uniform, frame-based operational definition of transient two-focus episodes. In a prophase-arrest reference, Cdc14–mCherry formed a predominant single nucleolar focus with occasional, reversible two-focus episodes that Nop56–GFP placed within the nucleolar compartment (nucleolar splitting). Splitting rose sharply when interhomolog recombination was compromised and remained elevated when Spo11 catalytic activity was abolished, indicating that increased DSB formation is not required and pointing instead to the homolog engagement state as a key variable. Population checkpoint readouts did not map onto the phenotype: Hop1 phosphorylation differed strongly across genotypes, yet splitting remained high in recombination-defective and DSB-free contexts and low in the reference. Timing analyses showed that events concentrated early and declined in the reference, whereas recombination-defective and DSB-free backgrounds retained activity into later windows across thresholds. We propose that nucleolar splitting reflects a rheological response of the nucleolus to chromosome-scale forces that vary with homolog engagement, consistent with contributions from DSB-independent chromosome dynamics such as telomere clustering, telomere-led rapid prophase movements, and centromere coupling/pairing. Together, these data support the nucleolus as a mesoscale, mechanically sensitive readout of meiotic chromosome dynamics.

## 1. Introduction

Meiosis establishes haploidy through a coordinated prophase I in which homologous chromosomes approach one another, assemble synaptonemal complex (SC), and exchange DNA. Double-strand breaks (DSBs) introduced by Spo11 initiate interhomolog recombination, yet only a subset of intermediates mature into crossovers (COs) that obey assurance and interference, implying long-range communication along chromosome length [1,2,3].

A convergent view is that key aspects of meiotic patterning arise from mesoscale assemblies that form on chromosome axes. Pro-crossover factors can assemble into droplet-like clusters whose number and spacing are shaped by coarsening and growth-competition dynamics. These behaviours offer a physical basis for crossover interference and assurance. Quantitative imaging and theoretical work support a model in which diffusion and local aggregation along synapsed chromosomes drive the emergence of these patterns [4]. In parallel, conserved regulators diffuse within the SC at rates compatible with transmitting regulatory information over chromosomal distances, and wetting-like assembly of the SC central region can organize axis-associated compartments and their mechanical couplings [5,6,7,8].

At the break-formation step, the Rec114–Mei4–Mer2 (RMM) module forms DNA-bound condensates with liquid-like properties consistent with phase separation. Tuning their material properties modulates Spo11-dependent DSB output, placing phase separation at the heart of meiotic DSB biogenesis [9]. Within this framework, processed DSBs generated in these assemblies recruit the ATR orthologue Mec1, which phosphorylates the axis protein Hop1 to trigger the recombination checkpoint and modifies Zip1 at centromeres to dismantle non-homologous centromere coupling [10,11,12]. The meiosis-specific recombinase Dmc1 then acts on resected Spo11-dependent breaks to promote interhomolog repair, linking DSB formation to both checkpoint signalling and engagement between homologues [13,14]. As interhomolog repair progresses and Mec1-dependent checkpoint signalling is relieved, transcriptional induction of *NDT80* initiates exit from prophase I. Ndt80 activates a middle meiotic gene-expression programme that coordinates late-prophase transitions, including shutdown of Spo11-dependent break formation, SC disassembly, and resolution of recombination joint molecules into COs, thereby bringing the homolog-engagement programme to completion and licensing progression beyond pachytene [15,16]. Collectively, these condensates, assembled on chromosomal cores, therefore reshape the mechanical, diffusive, and signalling landscape in which chromosome pairing and recombination unfold.

Chromosome motion is an active contributor. Telomere-led rapid prophase movements (RPM) and transient telomere clustering into the *bouquet* configuration transmit cytoskeletal forces to chromosome ends through the LINC axis (Ndj1–Mps3–Csm4 and associated bridges), combining nuclear rotation with directed telomere trajectories to remodel chromosome territories [17,18,19]. Early in prophase I, Zip1-dependent non-homologous centromere coupling and subsequent homolog-centric arrangements provide alternative attachment topologies that can either facilitate or hinder productive pairing; SC components can persist at centromeres after arm disassembly in some contexts [20]. These DSB-independent features—*bouquet*, RPM and centromere coupling/pairing—set attachment geometry and driving forces for whole-chromosome motion, thereby shaping the nuclear mechanical environment in which recombination proceeds [12,17,20,21,22].

The nucleolus is a multilayered biomolecular condensate. In budding yeast, the rDNA polymer together with a Cdc14-enriched chromatin domain defines a polymer–polymer phase-separated (PPPS) body that coexists with liquid–liquid phase-separated (LLPS) ribonucleoprotein layers formed by box C/D snoRNP components such as Nop56, which mark the bulk nucleolus; these subcompartments differ in rheology and coarsening behaviour and re-establish architecture rapidly after deformation [23,24,25,26]. In this framework, the nucleolus is expected to be sensitive to chromosome-borne forces and boundary conditions. Moreover, because the rDNA array on chromosome XII does not assemble canonical SC, forces routed through telomeres and centromeres can act on the nucleolar territory without SC reinforcement, making nucleolar organization a mesoscale reporter of the prophase I mechanical landscape [20,27,28,29,30].

Cdc14 is a conserved master cell-cycle phosphatase that undergoes regulated nucleolar sequestration and release to drive late mitotic and meiotic transitions. In budding yeast, nucleolar retention through the RENT complex (Cdc14–Net1–Sir2) and stage-specific release waves promote mitotic exit and accurate chromosome segregation [31,32,33,34,35,36,37]. Genetic analyses, including work from this laboratory, linked Cdc14 activity to the processing of meiotic recombination intermediates and to the fidelity of meiotic chromosome segregation, suggesting that spatial control of the rDNA-associated pool is functionally coupled to meiotic recombination dynamics [16,26]. This connection motivates two related questions. First, does the recombination and homolog-engagement context imprint nucleolar organization of the Cdc14 pool during prophase I? Second, can a defined, reversible redistribution within the nucleolus report the balance between DSB-dependent programmes and DSB-independent drivers of pairing/synapsis, as well as its relationship to population-level checkpoint readouts?

Here we confront a central gap. Modern work has established that meiotic recombination is organized by condensates that coarsen along the SC, that DSB output is modulated by condensate material properties, and that whole-chromosome motion is actively driven through telomere-clustering into the *bouquet* configuration, telomere-led RPM and centromere-based attachments. Yet a direct link between these programmes and the morphodynamics of the largest nuclear condensate, the nucleolus, under matched prophase I conditions is missing. We therefore ask whether a defined, reversible redistribution within the nucleolus can operate as a mechanically sensitive readout of the pairing/engagement landscape, and how such a readout relates to absolute DSB burden and to bulk checkpoint readouts, and whether it can be established rigorously with a uniform operational definition and an independent compartment marker. Resolving this point would bridge molecular regulation, mesoscale mechanics, and nuclear organization in prophase I, turning a longstanding descriptive gap into a tractable, quantitative question.

## 2. Results

### 2.1. A Recombination-Competent Prophase I Reference Uncovers Nucleolar Cdc14 Dynamics

**Figure 1:**
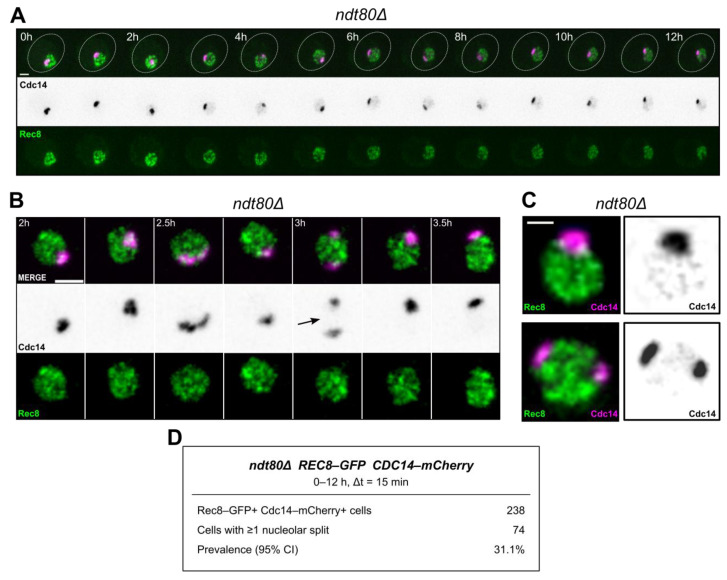
Prophase I reference in *ndt80*Δ: Cdc14 nucleolar distribution with occasional two-focus episodes. (**A**) Representative time-lapse of an *ndt80*Δ cell within the Rec8–GFP-gated prophase I window, illustrating the predominant pattern of a single bright Cdc14–mCherry focus within the nucleolar territory throughout the acquisition. Scale bar, 2 µm; frame interval (Δt), 15 min. (**B**) Short frame series from an independent *ndt80*Δ time-lapse illustrating a transient two-focus episode. The frame in which the two-focus configuration meets the pre-defined scoring criteria (local intensity minimum between peaks and centroid separation above the distance threshold, evaluated per 15 min frame) is marked with an arrow. Scale bar, 2 µm; frame interval (Δt), 15 min. Imaging conditions were matched to those in panel A (Section 4). (**C**) Frames from an independent *ndt80*Δ time-lapse series, showing a frame with a single nucleolar Cdc14–mCherry focus and a subsequent frame with a two-focus configuration that meets the predefined scoring criteria. Scale bar, 2 µm; frame interval (Δt), 15 min. Imaging conditions were matched to those in panel A (Section 4). (**D**) Summary table for the *ndt80*Δ reference dataset. For each time-lapse series, the table lists total duration and frame interval (Δt), together with the number of Rec8–GFP-gated prophase I cells scored under these criteria, the subset of cells that displayed at least one two-focus episode within the nucleolar territory, and the corresponding prevalence expressed as a percentage of gated cells.

Building on our previous findings [16], which linked Cdc14 to meiotic recombination, here we examined the converse question: whether the recombination context shapes the nucleolar distribution of Cdc14 during prophase I. Using time-lapse microscopy under matched scoring conditions, we analysed *ndt80*Δ to anchor all comparisons within prophase I and avoid confounding effects from progression into the meiotic divisions, when Cdc14 undergoes stage-specific relocalization. We restricted scoring to prophase I cells identified by Rec8–GFP, a meiosis-specific kleisin subunit of the cohesin complex that marks chromosome axes in prophase I [38,39,40]. In this reference background, Cdc14–mCherry accumulated as a bright focus within the nucleolar region (Figure 1A). Interestingly, in a subset of cells, the nucleolar signal transiently resolved into two spatially separated foci within the same territory and later re-joined within the same series, a dynamic behaviour that we scored as two-focus episodes within the nucleolar territory (Figure 1B,C, Video S1 and S2), using predefined criteria that required a clear intensity valley between two nucleolar Cdc14–mCherry maxima and a measurable spatial separation between their centroids in any 15 min frame, scored within a ≤12 h window to limit cumulative illumination (Section 4; Supplementary Figure S1). A representative wild-type time-lapse also showed transient nucleolar splitting of Cdc14 (Supplementary Figure S2), indicating that this behaviour is not confined to the *ndt80*Δ background. A compact summary of the number of prophase I cells scored under these criteria in the *ndt80*Δ reference is provided in Figure 1D. Thus, in a recombination-competent prophase I background, that executes Spo11-dependent break formation and early recombination steps but is prevented from progressing into the meiotic divisions, Cdc14 concentrated as a predominant single nucleolar focus with occasional two-focus episodes.

### 2.2. Recombination Failure Increases the Two-Focus Cdc14 Behaviour

**Figure 2:**
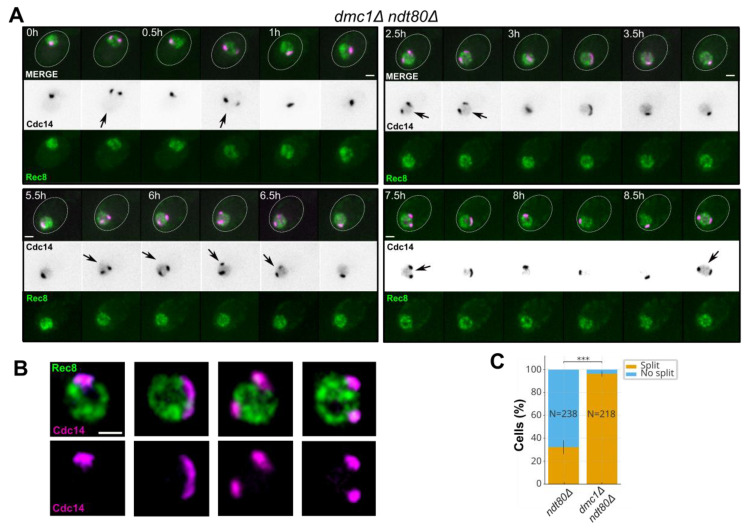
Recombination failure (*dmc1*Δ *ndt80*Δ) increases the two-focus Cdc14 behaviour. (**A**) Representative time-lapse of a *dmc1*Δ *ndt80*Δ prophase I cell (Rec8–GFP gate) showing frequent two-focus episodes of nucleolar Cdc14–mCherry within a single series. Arrows mark frames scored as two-focus episodes under the predefined criteria. Scale bar, 2 µm; frame interval (Δt), 15 min. (**B**) Representative still images of nucleolar Cdc14–mCherry under the same imaging conditions, illustrating cells with a single compact nucleolar focus and cells displaying two spatially separated nucleolar foci, to emphasize the intensity dip and spatial separation used in the event definition. Scale bar, 2 µm. (**C**) Stacked bar plot showing, for *ndt80*Δ and *dmc1*Δ *ndt80*Δ, the percentage of Rec8–GFP-positive (prophase I) cells that did or did not display at least one two-focus episode under the predefined scoring criteria (intensity dip plus centroid separation across 15 min frames within a ≤12 h window). Statistical significance is encoded by asterisks as follows: * *p* < 0.05; ** *p* < 0.01; *** *p* < 0.001; “ns” indicates *p* ≥ 0.05. Corresponding numerical values are summarized in Table 1. Acquisition settings were matched to those used in Figure 1.

Prompted by the initial observation of a two-focus redistribution of Cdc14, we next asked whether this behaviour is influenced by three features of the meiotic recombination landscape: interhomolog recombination, the accumulation of unrepaired meiotic DSBs, and the outcome of homology search. To isolate a condition in which these three dimensions are simultaneously impacted, we examined *dmc1*Δ *ndt80*Δ, in which the meiosis-specific recombinase Dmc1 is absent. Time-lapse series in this background showed that the nucleolar Cdc14–mCherry signal frequently resolved into two spatially separated foci within single cells (Figure 2A–C; Video S3). Quantification restricted to prophase I cells identified by Rec8–GFP indicated a marked increase in the fraction of cells that displayed the two-focus Cdc14 behaviour relative to the *ndt80*Δ reference (Table 1).

Event prevalence was defined as the fraction of cells with ≥1 nucleolar splitting episode over the 0–12 h time-course under Rec8–GFP prophase I gating (Section 4.4).

The same prophase I gating and event definition as in Section 2.1 were used (Figure 2B,C; Section 4; Supplementary Figure S1), ensuring that differences reflected genotype rather than scoring. Recurrent two-focus episodes were readily observed within single cells in *dmc1*Δ *ndt80*Δ (Figure 2A–C).

### 2.3. Nucleolar Splitting of Cdc14 Reveals a Dynamic Nucleolar Territory

**Figure 3:**
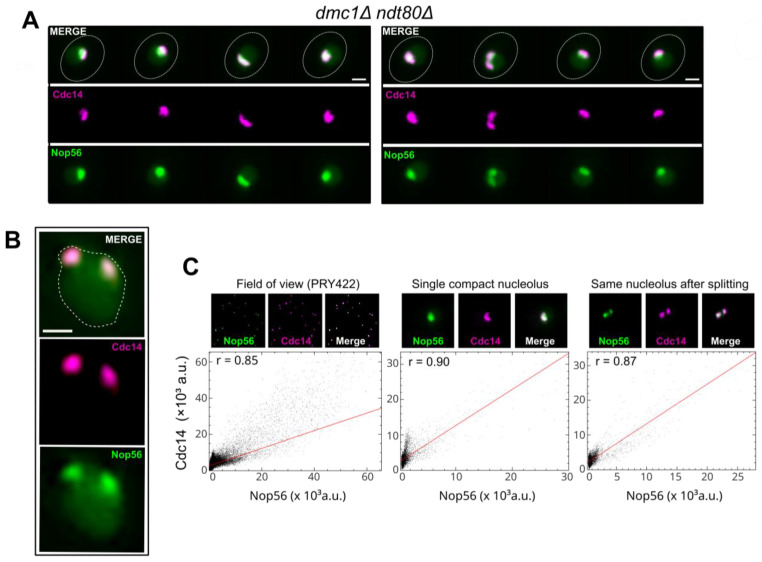
The two-focus Cdc14 behaviour maps to the nucleolus: coincidence with Nop56–GFP. (**A**) Representative time-lapse of a *dmc1*Δ *ndt80*Δ meiotic cell co-expressing Nop56–GFP and Cdc14–mCherry. The Cdc14–mCherry signal undergoes a transition from a single compact nucleolar focus to two spatially separated foci that remain confined within the Nop56–GFP-defined nucleolar territory before re-fusing. Micrometric scale bars (2 µm) and frame interval are indicated. (**B**) Single-frame example from a *dmc1*Δ *ndt80*Δ nucleus co-expressing Nop56–GFP and Cdc14–mCherry. The diffuse Nop56–GFP signal outlines the bulk nucleolus and extends slightly into the nucleoplasm; a sketched line indicates the predicted nuclear periphery, while Cdc14–mCherry appears in a two-focus configuration within the same nucleolar region, with an intensity valley separating the two peaks. (**C**) Representative pixel-intensity scatter plots (Cdc14–mCherry versus Nop56–GFP) and the corresponding images used for the analysis, illustrating the degree of spatial co-localisation during two-focus episodes. Pearson correlation coefficients for individual series are indicated for each scatter plot.

To determine whether the two-focus behaviour of Cdc14 reflects a property of the nucleolus rather than a fluorophore-specific artefact or a Cdc14-restricted redistribution, we analysed strains producing Nop56–GFP and Cdc14–mCherry within the same *ndt80*Δ-based time window used for prophase I scoring in the single-nucleolar-label experiments, applying the same predefined event criteria. *NOP56* encodes a core component of box C/D snoRNPs that marks the bulk nucleolus; its multivalent interactions support condensate formation within nucleolar subdomains (liquid–liquid/polymer–polymer phase separation) [25], providing a compartmental reference independent of Cdc14. In this dual-nucleolar labelling setting (Nop56–GFP plus Cdc14–mCherry), two-focus episodes of Cdc14 remained confined to the Nop56-defined nucleolar territory and showed measurable temporal coincidence with Nop56–GFP (Figure 3A–C; Video S4). Pixel-by-pixel intensity scatter plots confirmed this co-variation: a representative field of view showed a high Pearson correlation (r ≈ 0.85), a compact single nucleolus reached r ≈ 0.90, and the same nucleolus after a splitting episode still displayed a similarly high coefficient (r ≈ 0.87; Figure 3C). These examples indicate that Cdc14 largely remains within the Nop56-marked compartment even when the nucleolus adopts a two-focus configuration.

Because simultaneous imaging of two nucleolar fluorophores increases light dose, a factor known to perturb biomolecular condensates under intensive or prolonged illumination [41,42], analyses here were limited to compartment identity and temporal coincidence using the same event definition; absolute frequencies were not contrasted to the Rec8–GFP/Cdc14–mCherry datasets used for genotype comparisons, which were acquired with a lower nucleolar light budget. On this basis, we referred to this event as nucleolar splitting. Occasional divergences in timing or amplitude between Cdc14 and Nop56 signals were noted in individual series (Video S4) and are consistent with distinct partitioning across nucleolar subdomains, without altering the compartment-level conclusion [25].

### 2.4. Elevated Nucleolar Splitting Persists Without Meiotic DSB Formation

**Figure 4:**
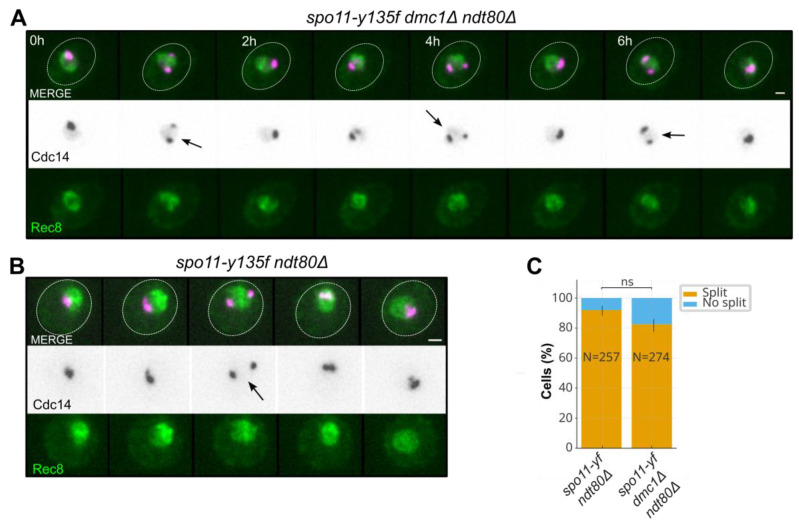
Elevated nucleolar splitting persists without meiotic DSB formation. (**A**) Representative time-lapse of a *spo11-y135f dmc1*Δ *ndt80*Δ prophase I cell (Rec8–GFP gate) showing frequent nucleolar splitting episodes of Cdc14–mCherry (two-focus configurations within the nucleolar territory) in the absence of Spo11-dependent DSBs. Arrows mark frames scored as nucleolar splitting events under the predefined criteria. Scale bar, 2 µm; frame interval (Δt), 15 min. (**B**) Representative time-lapse of a *spo11-y135f ndt80*Δ prophase I cell (Rec8–GFP gate) imaged under the same conditions, illustrating that nucleolar splitting remains common even when Dmc1 is present and Spo11-dependent DSB formation is abolished. Arrows indicate frames scored as nucleolar splitting events. Scale bar, 2 µm; frame interval (Δt), 15 min. (**C**) Stacked bar plot showing, for *spo11-y135f ndt80*Δ and *spo11-y135f dmc1*Δ *ndt80*Δ, the percentage of Rec8–GFP-positive (prophase I) cells that did or did not display at least one nucleolar splitting episode under the predefined scoring criteria (intensity dip plus centroid separation across 15 min frames, within a ≤12 h window). Bars show counts with 95% confidence intervals as appropriate (Section 4.6). Statistical significance is encoded by asterisks as follows: * *p* < 0.05; ** *p* < 0.01; *** *p* < 0.001; “ns” indicates *p* ≥ 0.05. Corresponding numerical values are summarized in Table 1.

To determine whether the elevated nucleolar splitting observed in *dmc1*Δ *ndt80*Δ relative to *ndt80*Δ stems from the accumulation of unrepaired DNA double-strand breaks or from deficits in homolog engagement, we abolished meiotic DSB formation using *spo11-y135f*, an allele of *SPO11* that encodes a catalytically inactive Spo11 variant. We introduced *spo11-y135f* into *ndt80*Δ and *dmc1*Δ *ndt80*Δ, keeping the prophase I scoring window and the event definition constant, in strains carrying Rec8–GFP as a prophase I marker together with Cdc14–mCherry to visualize the nucleolar pool. Removing DSB formation eliminates the substrate for resection and presynaptic filament assembly, allowing us to isolate the contribution of Spo11-dependent DSB load. Time-lapse series showed that nucleolar splitting remained frequent in *spo11-y135f dmc1*Δ *ndt80*Δ and was also elevated in *spo11-y135f ndt80*Δ compared with the *ndt80*Δ reference (Figure 4A–C; Table 1).

Quantification of the prevalence of nucleolar splitting (fraction of Rec8–GFP-positive cells with ≥1 episode) confirmed that both *spo11-y135f* backgrounds showed levels well above *ndt80*Δ (Table 1), whereas the difference between *spo11-y135f ndt80*Δ and *spo11-y135f dmc1*Δ *ndt80*Δ was modest (Figure 4C; Table 1). These data indicated that DSB formation per se was not the main driver and that nucleolar splitting of Cdc14 can remain elevated even in the absence of Spo11-dependent DSBs.

### 2.5. Nucleolar Splitting Is Decoupled from the Activity State of the Meiotic Recombination Checkpoint

**Figure 5:**
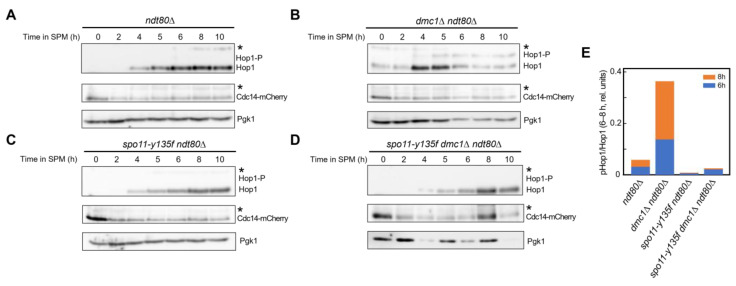
Population checkpoint readouts do not track nucleolar splitting. (**A**) Time-course immunoblot (0–10 h) for *ndt80*Δ showing Hop1, Hop1 phosphorylation (mobility-shifted band), Cdc14–mCherry and Pgk1 as loading control. (**B**) As in (**A**), for *dmc1*Δ *ndt80*Δ, illustrating strong and persistent Hop1 phosphorylation together with stable Cdc14–mCherry levels. (**C**) As in (**A**), for *spo11-Y135F ndt80Δ*, illustrating markedly reduced Hop1 phosphorylation across the time course in a background lacking Spo11-dependent meiotic DSB formation. (**D**) As in (**A**), for *spo11-Y135F dmc1*Δ *ndt80*Δ, showing a similarly low Hop1 phosphorylation pattern when both Spo11-dependent DSB formation and Dmc1 are absent. Cdc14–mCherry and Pgk1 signals are shown for all genotypes as controls for Cdc14 abundance and loading. (**E**) Quantification of Hop1 phosphorylation relative to total Hop1 at 6 and 8 h for the four genotypes. Bars represent average Hop1-P/Hop1 ratios calculated from these time points under the same culture and sampling conditions; corresponding numerical values and details of the quantification procedure are provided in Section 4.5. Asterisks depict unspecific bands in the blot.

Given that nucleolar splitting was observed both in a recombination-defective background with persistent checkpoint signalling (*dmc1*Δ *ndt80*Δ) and in DSB-free *spo11-y135f ndt80*Δ strains where recombination-checkpoint activation is not expected, we next examined more explicitly whether nucleolar splitting parallels the activity state of the meiotic recombination checkpoint at the population level. Population immunoblots for Hop1 phosphorylation and Cdc14–mCherry abundance were acquired on matched 0–10 h time courses in the same *REC8-GFP CDC14-mCherry* backgrounds used for live imaging (*ndt80*Δ, *dmc1*Δ *ndt80*Δ, *spo11-y135f ndt80*Δ and *spo11-y135f dmc1*Δ *ndt80*Δ; see Section 4), with Pgk1 as loading control (Figure 5A–D). Hop1 phosphorylation was weak or transient in *ndt80*Δ and strong and sustained in *dmc1*Δ *ndt80*Δ, and essentially undetectable in *spo11-y135f* backgrounds, consistent with persistent recombination-checkpoint activation in *dmc1*Δ *ndt80*Δ and with the abolition of Spo11-dependent DSB formation in *spo11-y135f* strains. Quantification of Hop1 phosphorylation relative to total Hop1 at 6 and 8 h (Figure 5E) confirmed this pattern, with checkpoint activation high in *dmc1*Δ *ndt80*Δ, intermediate in *ndt80*Δ and minimal in *spo11-y135f* backgrounds. Cdc14–mCherry levels were broadly comparable across genetic backgrounds and time points (Figure 5A–D). Yet nucleolar splitting was high in both *dmc1*Δ *ndt80*Δ and *spo11-y135f* backgrounds and low in *ndt80*Δ (Table 1), indicating that, under these matched conditions, nucleolar splitting did not parallel Hop1 phosphorylation at the population level and behaved as a readout largely independent of meiotic recombination-checkpoint activity. Given the similar Cdc14–mCherry levels, the changes in nucleolar splitting were not explained by alterations in Cdc14 abundance.

### 2.6. Timing and Persistence of Nucleolar Splitting Across Recombination Contexts

**Figure 6:**
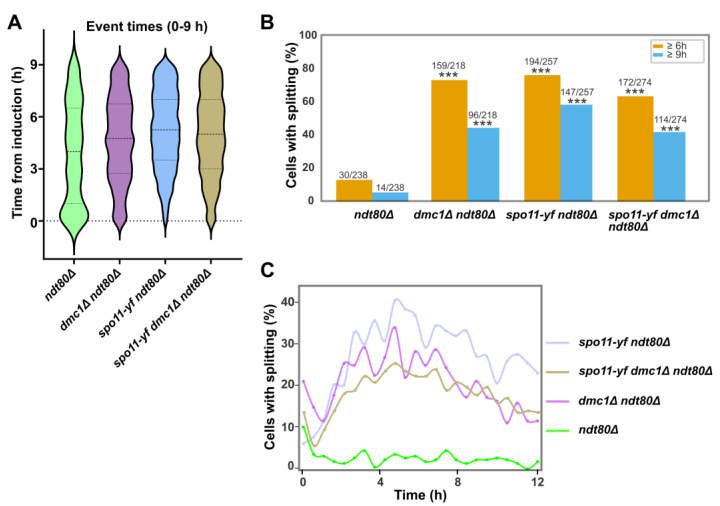
Temporal persistence of splitting across genetic backgrounds. (**A**) Violin plots showing the distribution of all nucleolar splitting event times (frames with ≥1 splitting event) from 0 to 9.0 h after induction for *ndt80*Δ, *dmc1*Δ *ndt80*Δ, *spo11-y135f ndt80*Δ and *spo11-y135f dmc1*Δ *ndt80*Δ. Central lines indicate median event times, with the width of each violin reflecting the density of events. Event-time quartiles (Q1/median/Q3, in hours) were 1.0/4.0/6.5 for *ndt80*Δ, 2.75/4.75/6.75 for *dmc1*Δ ndt80Δ, 3.5/5.25/7.0 for *spo11-y135f ndt80*Δ and 3.0/5.0/7.0 for *spo11-y135f dmc1*Δ ndt80Δ. The number of events contributing to each violin was 157, 1075, 1598 and 1173, respectively. (**B**) Fraction of cells with ≥1 nucleolar splitting event at or after 6.0 h and at or after 9.0 h for the same genotypes. Bars represent the percentage of Rec8–GFP-positive cells with at least one late event under each threshold, and labels above each bar indicate n/N cells with ≥1 late event. Asterisks on mutant bars denote significant increases relative to *ndt80*Δ at the same threshold (2 × 2 χ^2^ tests, df = 1; χ^2^ = 86.7–197.2, *p* < 10^−20^; full statistics in Table S2). (**C**) Temporal profiles of nucleolar splitting activity. Lines show the fraction of cells with ≥1 event per 0.5 h time bin over the 12 h time-course. Activity peaks early and remains low in *ndt80*Δ, whereas *dmc1*Δ and *spo11-y135f* backgrounds display broader, sustained activity extending into mid–late time bins. A χ^2^ test over the full binned time-course confirms a significant difference between *ndt80*Δ and *dmc1*Δ *ndt80*Δ, while *spo11-y135f ndt80*Δ and *spo11-y135f dmc1*Δ *ndt80*Δ are statistically indistinguishable (Table S2). TLAST summaries and Kruskal–Wallis tests for last-event times per cell are provided in Table S3.

To assess persistence rather than onset, we used three complementary timing metrics: (i) the distribution of individual event times scored within the 0–9.0 h window (Figure 6A), (ii) the fraction of cells with ≥1 event beyond predefined thresholds (6.0 h and 9.0 h; Figure 6B; Table S2), and (iii) for each cell, the time of its last observed event (TLAST; Table S3). The 6.0 h and 9.0 h thresholds were selected to balance late-time information with limits on cumulative illumination. The violin distributions of all events scored up to 9.0 h (Figure 6A) and the per-cell persistence table (Table S2) showed a graded pattern: in *ndt80*Δ the phenomenon concentrates early and wanes, whereas it remains frequent at late times in *dmc1*Δ and *spo11* contexts. Quantitatively, at 6.0 h (25 frames) late-event fractions were ~12.6% in *ndt80*Δ, ~72.9% in *dmc1*Δ *ndt80*Δ, ~75.5% in *spo11-y135f ndt80*Δ, and ~62.8% in *spo11-y135f dmc1Δ ndt80Δ,* supporting marked heterogeneity across genotypes; the same ordering was observed at the very-late 9.0 h cut-off (Figure 6B; Table S2). Two-by-two χ^2^ tests comparing each mutant to *ndt80*Δ at the same threshold confirmed that all contrasts were highly significant (χ^2^ = 86.7–197.2, df = 1, *p* < 10^−20^; full statistics in Table S2). Consistently, per-cell last-event medians (TLAST; Table S3) were earliest in *ndt80*Δ and shifted later in *dmc1*Δ and *spo11* backgrounds, consistent with non-parametric tests on TLAST distributions (Table S3). To avoid reliance on a single cut-off and to visualize when the population is active, a bin-based analysis (0.5 h bins; Figure 6C) showed that the proportion of cells with ≥1 event per bin peaks early and remained low in *ndt80*Δ, whereas *dmc1*Δ and *spo11* backgrounds displayed broader activity extending into mid–late time bins; a χ^2^ test over the full time-course confirmed a significant difference between *ndt80*Δ and *dmc1*Δ *ndt80*Δ, while *spo11-y135f ndt80*Δ and *spo11-y135f dmc1*Δ *ndt80*Δ were statistically indistinguishable (Table S2). Collectively, these timing analyses indicated that splitting persisted into late prophase in recombination-defective and DSB-free settings but declined in the *ndt80*Δ reference under identical scoring conditions. Complementary summary views of timing and persistence are shown in Supplementary Figure S3.

## 3. Discussion

We identify a transient two-focus redistribution of Cdc14 within the nucleolus during meiotic prophase I and show that its prevalence varies with the recombination landscape under a common prophase-anchored scoring regime. The behaviour is infrequent in a recombination-competent reference (*ndt80*Δ), increases when recombination fails (*dmc1*Δ), and remains elevated when Spo11-dependent DSB formation is abolished (*spo11-y135f*). Validation with Nop56-GFP places the event within the nucleolar compartment, and genotype-matched acquisition/analysis rules support that these differences reflect biology rather than imaging bias.

We use the term “nucleolar splitting” strictly for two-focus transitions that satisfy the predefined intensity-dip and separation criteria (Section 4), and only after compartment validation with Nop56-GFP. The term is not intended to imply rDNA breakage or irreversible nucleolar disruption; it denotes a reversible partition of the nucleolar signal, consistent with condensate-like behaviour [25,43].

Against this background, we next asked whether bulk recombination-checkpoint readouts track the phenomenon at the population level. Population checkpoint readouts do not map onto the pattern of nucleolar splitting. Strong Hop1 phosphorylation in *dmc1*Δ, weak or transient phosphorylation in *ndt80*Δ, and undetectable phosphorylation in *spo11–y135f* coexist with high splitting in *dmc1*Δ and *spo11–y135f* and low splitting in *ndt80*Δ (Figure 5A,B; Table 1; see also Figure 2 and Figure 4, and Figure 6A). Under the present scoring conditions, the phenomenon appears decoupled from recombination-checkpoint activity at the population level [10,44,45].

Although bulk checkpoint markers do not track the behaviour, an upstream ATR/ATM input may still contribute in specific contexts. During persistent Mec1 activation, as in the *dmc1*Δ background, Mec1-dependent phosphorylation of Zip1 at centromeres has been shown to dismantle Zip1-mediated centromere coupling [12,46]. Loss of coupling has been associated with altered whole-chromosome dynamics, which in our framework could facilitate force transmission to the rDNA and thereby be compatible with elevated nucleolar splitting. We therefore view this pathway as one plausible contributing route rather than a demonstrated mechanism in this study or a sole driver.

In *spo11–y135f*, a complementary DSB-independent route is plausible. Zip1-mediated centromere coupling and pairing can persist and may modulate force routing in the absence of breaks, while telomere *bouquet* formation and telomere-led RPM proceed through the Ndj1–Mps3–Csm4 LINC pathway: *bouquet* sets the attachment geometry, RPM supplies the driving forces. Together, these features could increase the degrees of freedom of whole-chromosome motion and facilitate force transfer to the rDNA [17,20,21], consistent with the persistence of nucleolar splitting in this context. In future work, genetic perturbations that differentially affect telomere attachment versus force production could be used to challenge this model.

We interpret the two-focus behaviour as more than a passive consequence of bulk chromosomal motion. The Nop56–GFP dataset shows that the event involves the nucleolus as a compartment, while occasional divergences from Cdc14–mCherry indicate that nucleolar subdomains need not move in perfect register. Work in mitotic cells already supports a mixed-condensate architecture at the rDNA locus. Quantitative imaging showed that Cdc14 and other rDNA-bound factors occupy a compressible polymer condensate, whereas Nop56-marked ribonucleoproteins form a more homogeneous, liquid-like phase that neither compacts when rDNA loops contract nor disperses when the rDNA array is lost [25]. In physical terms, the nucleolus behaves as a biomolecular condensate [24,47]; transient splitting then reflects a rheological response of that condensate to chromosome-borne forces when homolog engagement is compromised or delayed [17,48,49]. The rapid return to a single body is naturally explained by coalescence/coarsening dynamics once the deforming constraint relaxes [6].

This framework is consistent with our genetics. Elevated splitting in *dmc1*Δ and in *spo11–y135f*—despite opposite DSB contexts—implicates the state of homolog engagement rather than DSB load as the decisive variable [50]. In budding yeast, the rDNA array on chromosome XII does not assemble synaptonemal complex [20,27,28,29,30]; accordingly, forces arising from pairing attempts and centromere/telomere reorganizations during prophase I can act without SC reinforcement at the rDNA, making the nucleolus a sensitive readout of that mechanical landscape [17,20,48]. The lack of correspondence with checkpoint readouts further supports a mechanical, rather than signalling-driven, origin under our conditions [10].

Finally, the modest desynchrony sometimes observed between Cdc14–mCherry and Nop56–GFP fits a model in which Cdc14-rich and Nop56-rich subcompartments differ in composition and relaxation times (liquid-like versus more polymer-dominated features), allowing small phase-specific offsets in their motions without altering the compartment-level conclusion [25]. In sum, our observations support that “nucleolar splitting” behaves as a mesoscale, threshold-dependent mechanical readout of prophase I chromosome dynamics, and its fast resolution reflects the material properties of the nucleolus itself. DSB-independent routes of homolog proximity may contribute to this picture. Prior work described non-homologous centromere coupling in early prophase I and its dependence on the synaptonemal complex component Zip1, which can organize centromeres in the absence of Spo11-generated breaks [12,20,46]. Such coupling may provide ordered coalescence of centromere-associated factors and, in pathological regimes, promote aggregate-like assemblies [51,52,53]. Within this framework, Zip1-mediated centromere coupling in *spo11–y135f* could sustain pairing attempts and associated force transmission even without DSBs, consistent with transient partitioning of the nucleolar condensate; coarsening could rapidly restore a single body. This hypothesis is consistent with the elevated splitting observed in *spo11–y135f* and can be further refined by future genetic dissection of centromere-coupling pathways [12].

**Figure 7:**
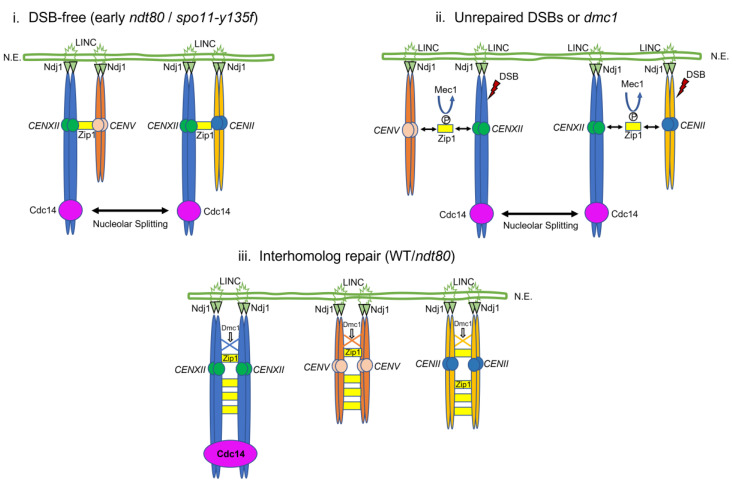
Working model linking recombination status, telomere-driven forces, and nucleolar splitting. (**i**) DSB-free (early *ndt80*Δ/*spo11-y135F*). Telomeres of homologous chromosomes are anchored to the nuclear envelope (N.E.) through Ndj1–LINC complexes. Before Spo11 dependent DSB formation, Zip1 accumulates at centromeres and promotes non-homologous centromere coupling (examples shown for *CENXII* and *CENV*, and for *CENXII* and *CENII*). Under this configuration, rapid prophase movements transmitted from Ndj1–LINC to poorly engaged homologs could deform the rDNA bearing region and be compatible with dynamic nucleolar splitting, visualized as oscillation of Cdc14 between two separated nucleolar bodies. (**ii**) Unrepaired DSBs or *dmc1*Δ. Spo11 induced DSBs can activate Mec1; Mec1-dependent phosphorylation of centromeric Zip1 has been shown to release non-homologous centromere couplings. In *dmc1*Δ, or in early *ndt80*Δ before interhomolog repair completes, homolog engagement remains defective despite centromere decoupling. Telomere-driven forces persist in this configuration and continue to pull on incompletely paired chromosome XII arms, so nucleolar splitting remains frequent and long lived. (**iii**) Interhomolog repair (WT/*ndt80*). When Dmc1 driven interhomolog repair and synaptonemal complex assembly are established, homologous centromeres and arms are fully engaged by Zip1. Ndj1–LINC still anchors telomeres, but forces are expected to be buffered by a rigid bivalent, consistent with stabilization of the nucleolar condensate and Cdc14 concentrates in a single nucleolar body with rare splitting episodes. In all panels, only one telomere attachment per homolog and a subset of centromeres are shown for simplicity; Ndj1–LINC anchoring is assumed to operate in all genotypes.

These elements are summarized in a working model (Figure 7) that links recombination status, homolog engagement, and telomere-driven forces to nucleolar behaviour. In a DSB-free configuration (very early *ndt80*Δ or *spo11-y135F*), telomeres anchored via Ndj1–LINC and Zip1-mediated centromere coupling may act on poorly engaged chromosome XII arms, consistent with repeated splitting of the Cdc14/Nop56 condensate. When Spo11-dependent DSBs form but interhomolog repair is compromised (for example in *dmc1*Δ *ndt80*Δ), Mec1-dependent regulation of centromeric Zip1 has been shown to release non-homologous couplings, but homolog engagement remains defective, and telomere-led forces could still promote splitting of the nucleolar condensate. Once Dmc1-dependent interhomolog repair and ZMM/SC assembly establish robust homolog engagement (in recombination-competent settings, including late *ndt80*Δ cells), telomere attachments persist but forces are expected to be buffered by paired bivalents and nucleolar splitting could subside.

Together, the data and framework converge on a simple working view: under matched scoring conditions, nucleolar splitting tracks the state of homolog engagement more than the absolute burden of Spo11-dependent DSBs. The checkpoint does not map onto the behaviour at the population level under our conditions, whereas routes that may alter attachment/force routing (centromere coupling via Zip1; telomere-led RPM via the Ndj1–Mps3–Csm4 LINC axis) offer specific, testable predictions for late-time persistence profiles. A next step will be to define how Cdc14 responds to these mechanical inputs in real time, and whether its nucleolar redistribution has consequences for recombination factors at chromosome arms, centromeres, or the rDNA-proximal region. Such experiments may reveal additional modes of Cdc14 control over meiotic recombination that are not apparent from bulk checkpoint readouts alone.

## 4. Materials and Methods

### 4.1. Yeast Strains and Media

All experiments used *Saccharomyces cerevisiae* SK1 background. Datasets underlying Section 2.1, Section 2.2, Section 2.4, Section 2.5 and Section 2.6 carried Rec8–GFP or Zip1-GFP as a nuclear/chromosome marker together with Cdc14–mCherry to visualize the nucleolar Cdc14 pool. The dual-nucleolar validation in Section 2.3 employed Nop56–GFP plus Cdc14–mCherry and did not include Rec8–GFP. Full genotypes are provided in Table 2. Standard YPD, pre-sporulation, and sporulation media were used as summarized in Supplementary Table S4.

### 4.2. Meiosis Induction and Time Courses

Cells were grown in pre-sporulation medium, shifted to sporulation medium, and incubated at 30 °C. Time courses were initiated upon transfer to sporulation medium. For live-cell imaging, time-lapse acquisitions were initiated at approximately 5 h into the time course: a 75 µL sample was collected and diluted in fresh sporulation medium to a final volume of 1 mL (3/40 dilution) to reach the cell density used for microscopy, and 300 µL of the diluted culture was loaded into each microscopy chamber. Time-lapse series were acquired at 15 min per frame and typically extended up to ~12 h. Analyses were restricted to prophase I cells gated by Rec8–GFP, or Zip1-GFP, where applicable. Acquisition windows and thresholds used to limit cumulative light exposure are specified below and in Section 2.6.

### 4.3. Live-Cell Microscopy and Acquisition Parameters

Time-lapse imaging was performed on a spinning-disk confocal platform Roper Scientific with Olympus IX81 inverted microscope (Olympus Corporation, Tokyo, Japan); the optical components employed was the 100× 1.4 NA objective and an Evolve (Photometrix, Tucson, AZ, USA) camera. For each time point, a short Z-stack was acquired (typically 11 planes with a Z-step of 0.4 µm, spanning ~4 µm in depth) around the brightfield focal plane, which approximates the mid-plane of most cells in the field. Under these conditions, the nuclear volume was generally encompassed within the imaged Z-range. Z-stacks were rendered by maximum-intensity projection with fixed LUTs and exposure ranges within each genotype-matched experiment. Excitation, exposure, EM gain/laser power, Z-step and frame size were kept constant per dataset; numerical ranges are reported in Table 3 and Table S1.

Time-lapse series in which the Cdc14–mCherry or Nop56–GFP nucleolar signal approached or touched the top or bottom limits of the stack, or appeared clearly truncated in Z, were excluded from analysis.

### 4.4. Image Processing, Gating and Event Definition

Raw image stacks were processed in Fiji (version 1.54)/MetaMorph (version 7.10.5 release 2022) using a fixed pipeline: drift correction when needed, projection, background estimation from local annuli, and nucleolar ROI tracking from the Cdc14–mCherry signal. Prophase I gating relied on Rec8–GFP in single-nucleolar datasets. A “two-focus event” was scored in any 15 min frame in which the Cdc14–mCherry signal within the nucleolar ROI displayed two intensity maxima separated by a clear local minimum and a measurable distance between their centroids, with both foci fully contained within the imaged Z-stack; frames were scored only within the first 12 h of the time course to limit cumulative illumination. Exact thresholds, ROI geometry and exclusion rules are summarized in Supplementary Figure S1A. The term “nucleolar splitting” is used only after compartment validation with Nop56–GFP (Section 2.3) for events that meet these criteria. In dual-nucleolar datasets (Nop56–GFP + Cdc14–mCherry), coincidence was quantified within the Nop56-defined nucleolar territory using time-aligned overlays and line-profile analyses [54]; absolute frequencies were not contrasted to single-label datasets due to different illumination budgets. Visual inspection of representative raw stacks confirmed that these two-focus episodes reflect a physical partition of the nucleolar signal rather than incomplete sampling of elongated nucleolar profiles at the periphery of the Z-stack. For colocalization analyses of Nop56–GFP and Cdc14–mCherry (Figure 3C), maximum-intensity projections were generated for each channel and a nucleolar region of interest was defined as above. Pixel-wise colocalization within this region was quantified with the Coloc2 plugin (release 3.1.0) in Fiji, reporting the Pearson correlation coefficient r; representative scatter plots and r values are shown in Figure 3C.

### 4.5. Immunoblotting

Protein extracts were prepared by TCA precipitation [55,56], resolved by SDS–PAGE [57], transferred to PVDF and probed with antibodies listed in Table 4. Time courses covered 0–10 h under the same sporulation regime used for imaging. All time courses were performed in the *REC8–GFP CDC14–mCherry* strains used for live imaging in Figure 1, Figure 2, Figure 3, Figure 4 and Figure 6 (genotypes in Section 4.1.). Detection used standard ECL within the linear range; exposures were matched across genotypes within an experiment. Band intensities for Hop1, Hop1-P, Cdc14–mCherry and Pgk1 (Figure 5) were quantified from non-saturated exposures using Fiji. Rectangular regions of interest were drawn around each band, background was subtracted locally, and integrated intensities were exported to R. For each lane, the Hop1-P/Hop1 ratio was calculated and, where indicated, normalized to the mean *ndt80*Δ value at 6 h to obtain the relative checkpoint readouts plotted in Figure 5E.

### 4.6. Quantification and Statistics

Event prevalence was defined as the fraction of cells with ≥1 event per genotype; recurrence was the number of events per cell. Timing metrics included the distribution of all event times displayed as violin plots (0–9.0 h), the last observed event time per cell (TLAST, computed within a 0–12 h window), late-event fractions at or after 6.0 h and at or after 9.0 h, and 0.5 h bin profiles of the fraction of cells with ≥1 event over the 12 h time-course. Proportions were compared by χ^2^ tests with two-sided α = 0.05. 95% confidence intervals were obtained by bootstrap when appropriate. In all figures, statistical significance is encoded by asterisks as follows: * *p* < 0.05; ** *p* < 0.01; *** *p* < 0.001; “ns” indicates *p* ≥ 0.05. For prevalence measurements, exact *N*, *n* and percentage values are reported in Table 1. For persistence metrics (late-event fractions and TLAST), summary statistics and test values are provided in Supplementary Tables S2 and S3. Analyses were carried out in R (Core Team, version 4.5.2) and Fiji (ImageJ) or MetaMorph (Molecular Devices, San Jose, CA, USA), with scripts and macros available upon request.

### 4.7. Reporting and Data Availability

Figure panels show representative series acquired under genotype-matched settings; scale bars and time stamps are indicated where relevant. Source data (per-cell counts, event times, TLAST and binned activity curves), together with analysis scripts, will be provided in the Supplementary Materials (Supplementary Figure S1 and Supplementary Tables S2 and S3).

## Figures and Tables

**Table 1:** Prevalence of nucleolar splitting under Rec8–GFP prophase I gating.

Genotype	Total Cells (N)	Cells with ≥1 Splitting Event (n)	Prevalence (%)
*ndt80*Δ	238	74	31.1
*dmc1*Δ *ndt80*Δ	218	209	95.9
*spo11-y135F ndt80*Δ	257	236	91.8
*dmc1*Δ *spo11-y135f ndt80*Δ	274	225	82.1

**Table 2:** List of yeast strains used in this study.

Strain	Genotype
PRY414	*MAT a/α ho::LYS2/hisG, ndt80::hphMX6/″, CDC14-mCherry–ClonNat/″, REC8–GFP::LEU2::KanMX4/″, trp1/TRP1, his4X::LEU2 (NgoMIV; ori)–URA3/HIS4*
PRY221	*MAT a/α ho::LYS2/ho::hisG, ndt80::hphMX6/″, CDC14-mCherry–ClonNat/″, REC8–GFP::LEU2::KanMX4/″, his4/″, dmc1Δ::KanMX4/″, ura3/″*
PRY416	*MAT a/α ho::hisG/″, ndt80::hphMX6/″, CDC14-mCherry–ClonNat/″, REC8–GFP::LEU2::KanMX4/″, trp1/″, his4X::LEU2 (NgoMIV; ori)–URA3/″, arg4/″, spo11–Y135F–HA–URA3/″, dmc1Δ::KanMX4/DMC1*
PRY346	*MAT a/α, ho::LYS2, lys2, ndt80::hphMX6/″, CDC14-mCherry–ClonNat/″, REC8–GFP::LEU2::KanMX4/″, dmc1Δ::KanMX4/″, spo11–Y135F–HA–URA3/″, arg4/″, HIS4/his4*
PRY420	*MAT a/α NOP56–GFP::URA3, CDC14–mCherry::ClonNat, ndt80::hphMX6, leu2/″, lys2/″, his4/″, trp1/″*
PRY422	*MAT a/α NOP56–GFP::URA3, CDC14–mCherry::ClonNat, ndt80::hphMX6, dmc1Δ::KanMX4, leu2/″, lys2/″, his4/″, trp1/″*
PRY331	*MAT a/α ho::LYS2/”, his4X::LEU2(NgomIV;ori)-URA3/”, GFP(S65)-TUB1-URA3/”, CDC14-mcherry-ClonNat/”, ZIP1-GFP (at AA700)/”, arg4/ARG4*

**Table 3:** Channel-specific imaging settings.

Channel	Z-Stack	Exposure Time (ms)	Camera Readout (MHz)	Laser Power (% of max)	Analog Gain (×)	EM Gain
TRANS	No	–	5	–	3 (4×)	800
GFP	Yes (11 × 0.4 µm)	100	5	18	3 (4×)	800
mCherry	Yes (11 × 0.4 µm)	200	5	8	3 (4×)	800

Live-cell microscopy settings. Channel-specific exposure and laser settings on the EM-CCD.

**Table 4:** Antibodies used in this study.

Target	Antibody Description	Supplier	Dilution (WB)
Hop1	Mouse monoclonal anti-Hop1	Genescript (Piscataway, NJ, USA)	1:1000
RFP (Cdc14–mCherry)	Rat monoclonal anti-RFP (clone 5F8)	ChromoTek (Planegg-Martinsried, Germany)	1:2000
PGK1	Mouse monoclonal anti-PGK1 (clone 22C5)	Invitrogen (Carlsbad, CA, USA)	1:10,000
Mouse IgG (secondary)	Polyclonal anti-mouse IgG–HRP	Thermo Fisher Scientific (Waltham, MA, USA)	1:5000
Rat IgG (secondary)	Polyclonal anti-rat IgG–HRP	Thermo Fisher Scientific (Waltham, MA, USA)	1:5000

## Data Availability

The original contributions presented in this study are included in the article/Supplementary Material. Further inquiries can be directed to the corresponding author(s).

## Supplementary Materials

The following supporting information can be downloaded at: https://www.mdpi.com/article/10.3390/ijms27020888/s1.

## Abbreviations

The following abbreviations are used in this manuscript:

DSB	Double-strand break
SC	Synaptonemal complex
RPM	Rapid prophase movements
LLPS	Liquid–liquid phase separation
PPPS	Polymer-polymer phase separation
TLAST	Last observed nucleolar splitting event time per cell
RMM	Rec114-Mei4-Mer2 module
NE	Nuclear Envelope
