# Supplementary material for: Nucleolar Cdc14 Splitting Reflects Recombination Context and Meiotic Chromosome Dynamics
Source: Int J Mol Sci. 2026 Jan 15;27(2):888. doi: 10.3390/ijms27020888 (PMC12841278; doi:10.3390/ijms27020888)
Supplement: Supplementary file 1 [file ijms-27-00888-s001.zip › All_Supp_Tables.pdf]

Table S1. Z-stack and time-lapse acquisition parameters

Z-range (µm)	Section thickness (µm)	Number of sections	Number of positions	Number of time points
4.0	0.4	11	10	49

Z-range, optical section thickness, number of Z-sections, positions per field and total number of time points used for all live-cell time-lapse acquisitions (15-min intervals over a 12-h window).

Supplementary Table S2A. Late-event fractions at 6.0 h and 9.0 h under Rec8–GFP prophase-I gating.

Genotype	N cells	Cells with ≥1 event ≥6.0 h (n)	Fraction ≥6.0 h (%)	Cells with ≥1 event ≥9.0 h (n)	Fraction ≥9.0 h (%)
ndt80Δ	238	30	12.6	14	5.9
dmc1Δ ndt80Δ	218	159	72.9	96	44.0
spo11-Y135F ndt80Δ	257	194	75.5	147	57.2
dmc1Δ spo11-Y135F ndt80Δ	274	172	62.8	114	41.6

χ^2 tests comparing late-event fractions at 6.0 h and 9.0 h to the *ndt80Δ* reference.

Supplementary Table S2B.

Comparison vs *ndt80Δ*	Threshold (h)	χ^2 (df = 1)	p-value
dmc1Δ ndt80Δ	6.0	170.6	5.34×10^{-39}
spo11-y135f ndt80Δ	6.0	197.2	8.46×10^{-45}
dmc1Δ spo11-y135f ndt80Δ	6.0	134.2	4.94×10^{-31}
dmc1Δ ndt80Δ	9.0	90.5	1.86×10^{-21}
spo11-y135f ndt80Δ	9.0	148.3	4.14×10^{-34}
dmc1Δ spo11-y135f ndt80Δ	9.0	86.7	1.27×10^{-20}

Late-event fractions at 6.0 h and 9.0 h were compared to the *ndt80Δ* reference using 2×2 χ^2 tests (df = 1); all contrasts were highly significant ($p < 10^{-20}$).

Supplementary Table S3. Summary of last-event time (TLAST) per genotype under Rec8–GFP prophase-I gating.

Genotype	n cells with ≥1 event	TLAST median (h)	TLAST IQR (h; Q1–Q3)	Mean TLAST (h)	SD TLAST (h)
ndt80Δ	74	5.13	1.69–7.25	5.08	3.72
dmc1Δ ndt80Δ	209	8.50	6.25–10.50	8.12	2.79
spo11-Y135F ndt80Δ	236	10.25	7.44–11.50	9.16	2.83
spo11-y135f dmc1Δ ndt80Δ	225	9.00	6.25–11.00	8.37	2.90

TLAST was defined as the last frame (0–12 h, 15-min intervals) with a detectable nucleolar splitting event per cell. The table reports, for each genotype, the number of cells with ≥1 event, the median TLAST with interquartile range (IQR), and the mean ± SD.

Supplementary Table S4. Media

Media	Components
YEPD	Yeast extract (1%), Bacto-peptone (2%), glucose (2%)
YEPD agar	Yeast extract (1%), Bacto-peptone (2%), glucose (2%), agar (2%)
YPA	Yeast extract (1%), Bacto-peptone (2%), Potassium acetate (1%)
SPM	Potassium acetate (1%)
Minimal medium	Yeast Nitrogen Base (-aa) 0.7%, glucose 2% and agar 2%
