# Supplementary material for: Nucleolar Cdc14 Splitting Reflects Recombination Context and Meiotic Chromosome Dynamics
Source: Int J Mol Sci. 2026 Jan 15;27(2):888. doi: 10.3390/ijms27020888 (PMC12841278; doi:10.3390/ijms27020888)
Supplement: Supplementary file 1 [file ijms-27-00888-s001.zip › ijms-4045943-Supplementary Video Legends.pdf]

Supplementary Video Legends

Video S1: *ndt80Δ* time-lapse fields illustrating nucleolar compartment confinement of Cdc14 and occasional timing offsets between cells. Movies show multiple cells per field; the apparent frequency of splitting episodes in these examples is illustrative and does not necessarily match the exact prevalence values reported in the main figures. Scale bar 5 μm.

Video S2: *ndt80Δ* time-lapse fields illustrating nucleolar compartment confinement of Cdc14 and splitting episode in a single cell. It does not necessarily match the exact timing values reported in the main figures.

Video S3: *dmc1Δ ndt80Δ* time-lapse fields illustrating nucleolar compartment confinement of Cdc14 and more frequent splitting episodes with timing offsets between cells. Movies show multiple cells per field under the same imaging conditions as in Video S1; the apparent frequency of events is illustrative and not intended to reproduce a specific quantified dataset. Scale bar 5 μm.

Video S4: Additional fields with dual-nucleolar examples for Nop56–GFP/Cdc14–mCherry, illustrating that Cdc14-rich and Nop56-rich signals remain confined within the nucleolar compartment while occasionally resolving into two bodies. Movies show multiple nucleoli per field under the same conditions used for the Nop56–GFP/Cdc14–mCherry analyses. Scale bar 5 μm.
