# Supplementary material for: Nucleolar Cdc14 Splitting Reflects Recombination Context and Meiotic Chromosome Dynamics
Source: Int J Mol Sci. 2026 Jan 15;27(2):888. doi: 10.3390/ijms27020888 (PMC12841278; doi:10.3390/ijms27020888)
Supplement: Supplementary file 1 [file ijms-27-00888-s001.zip › All_Supp_Figures.pdf]

Figure S1

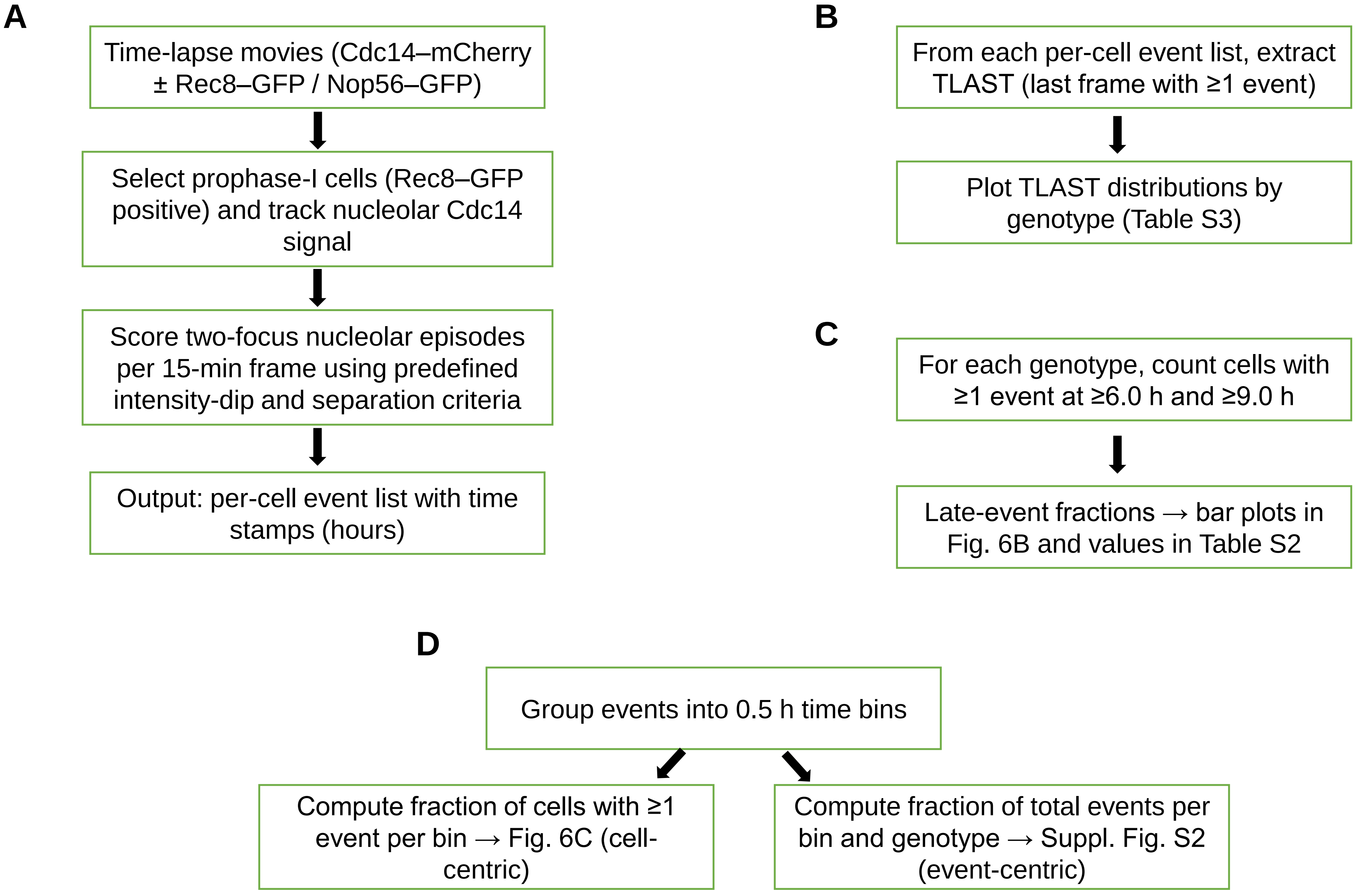
A
Time-lapse movies (Cdc14–mCherry ± Rec8–GFP / Nop56–GFP)
Select prophase-I cells (Rec8–GFP positive) and track nucleolar Cdc14 signal
Score two-focus nucleolar episodes per 15-min frame using predefined intensity-dip and separation criteria
Output: per-cell event list with time stamps (hours)
B
From each per-cell event list, extract TLAST (last frame with ≥1 event)
Plot TLAST distributions by genotype (Table S3)
C
For each genotype, count cells with ≥1 event at ≥6.0 h and ≥9.0 h
Late-event fractions → bar plots in Fig. 6B and values in Table S2
D
Group events into 0.5 h time bins
Compute fraction of cells with ≥1 event per bin → Fig. 6C (cell-centric)
Compute fraction of total events per bin and genotype → Suppl. Fig. S2 (event-centric)

Figure S1. Workflow for nucleolar splitting quantification and persistence metrics.
(**A**) From time-lapse series, Rec8–GFP-positive prophase-I cells are selected and the nucleolar region is tracked based on Cdc14–mCherry. Two-focus episodes are scored when the nucleolar Cdc14 signal resolves into two intensity maxima separated by a local minimum that satisfies predefined persistence and separation criteria (Methods). (**B**) For each cell, the times of all scored events are compiled and the last observed event time (TLAST) is extracted. (**C**) Late-event fractions are obtained as the proportion of cells with ≥ 1 event at or after 6.0 h and at or after 9.0 h under identical frame sampling across genotypes. (**D**) Population activity curves are generated by grouping events into 0.5 h bins and computing, for each bin, either the fraction of cells with ≥ 1 event (main-text Fig. 6C) or the fraction of total events per genotype (Supplementary Fig. S2). Together, these metrics provide complementary views of nucleolar splitting prevalence and temporal persistence under matched scoring conditions.

Figure S2

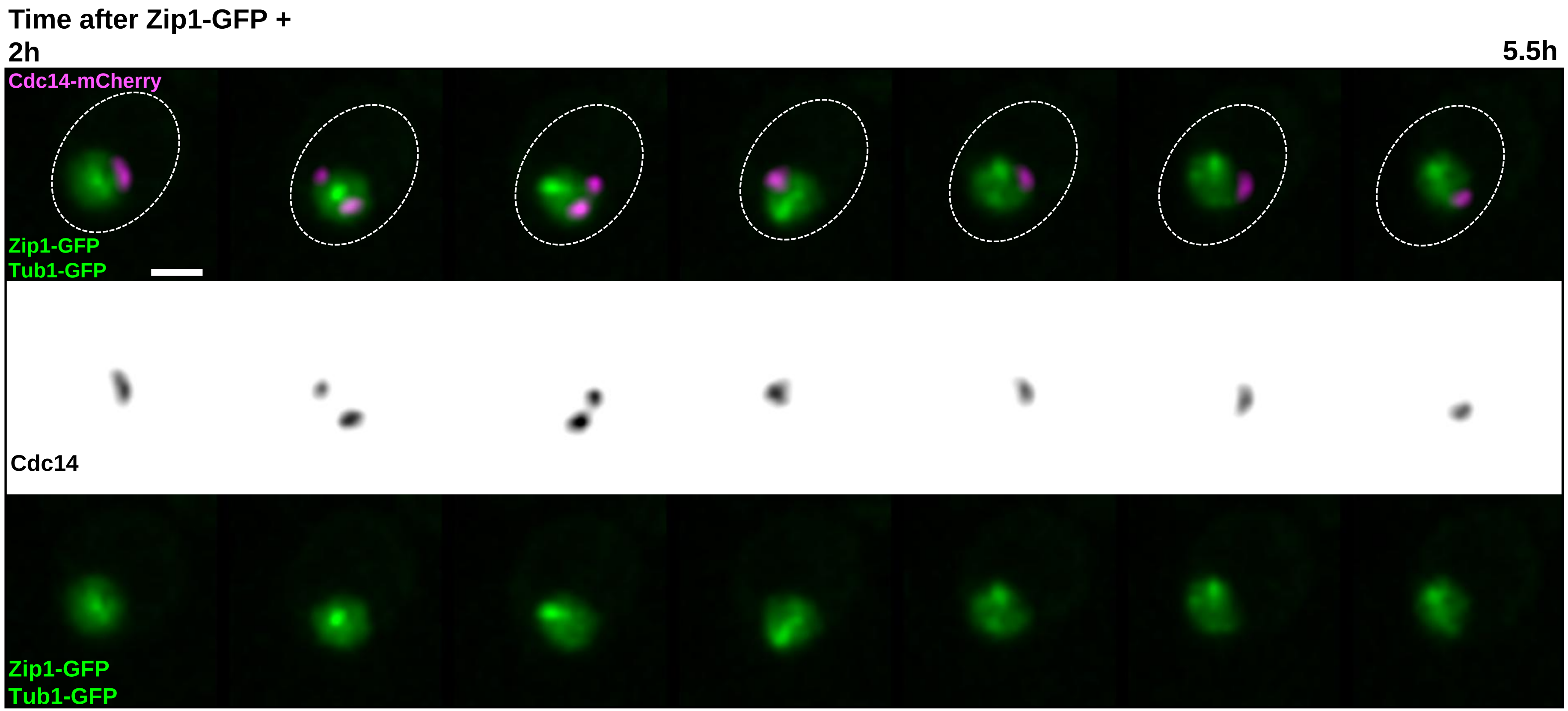

NDT80 DMC1 SPO11

Figure S2. Wild-type cells display transient nucleolar splitting of Cdc14. Representative live-cell time-lapse from a wild-type strain shows Cdc14–mCherry concentrating in the nucleolar region and undergoing a reversible transition into two spatially separated foci within the same nucleolar territory (“splitting”), followed by re-fusion within the same series. Time stamps are shown relative to first frame where Zip1-GFP assembly was noted; Scale bar, 2 µm.

Figure S3

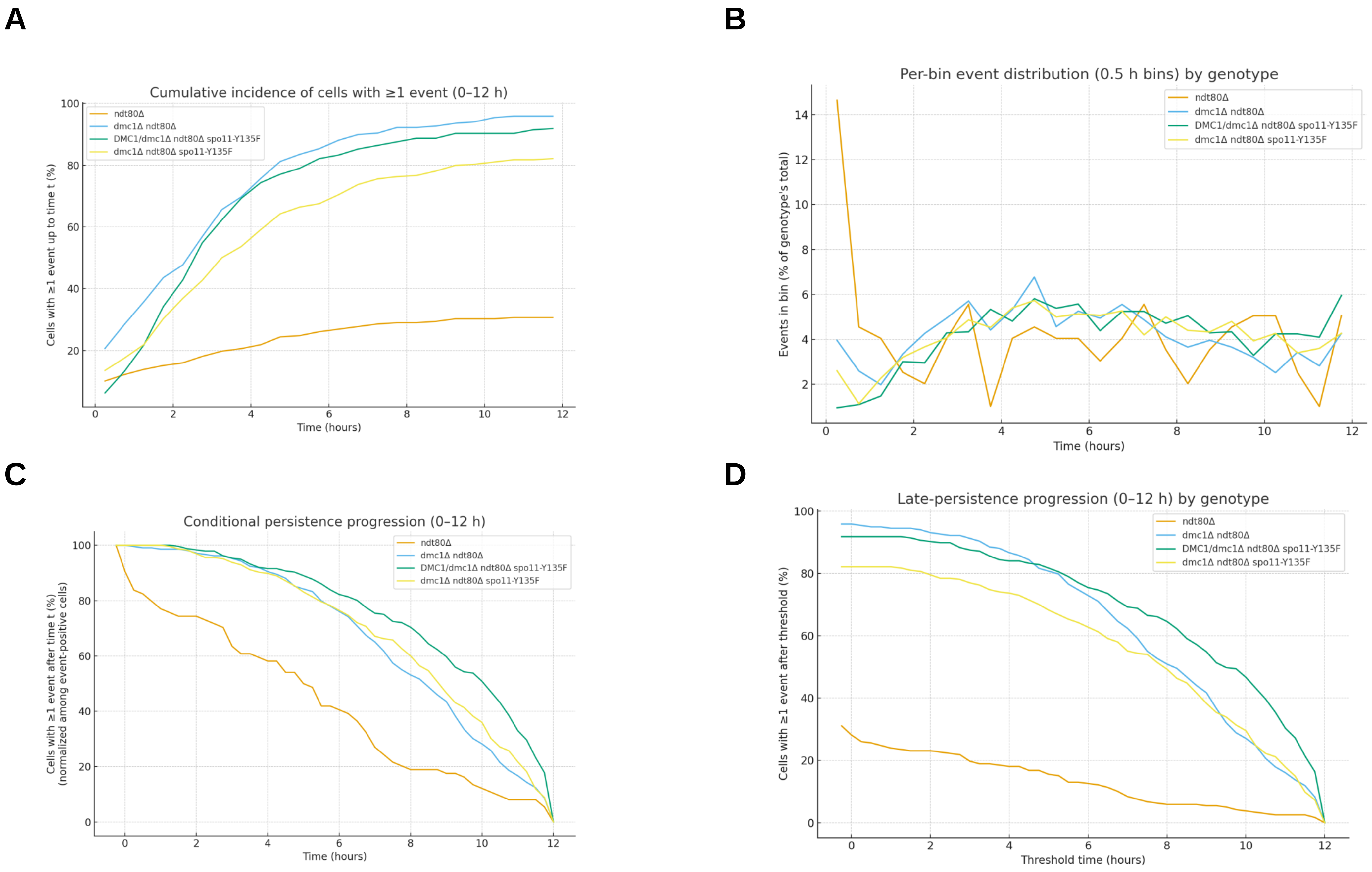
A
Cumulative incidence of cells with ≥1 event (0–12 h)
ndt80Δ
dmc1Δ ndt80Δ
DMC1/dmc1Δ ndt80Δ spo11-Y135F
dmc1Δ ndt80Δ spo11-Y135F
Cells with ≥1 event up to time t (%)
Time (hours)
B
Per-bin event distribution (0.5 h bins) by genotype
ndt80Δ
dmc1Δ ndt80Δ
DMC1/dmc1Δ ndt80Δ spo11-Y135F
dmc1Δ ndt80Δ spo11-Y135F
Events in bin (% of genotype's total)
Time (hours)
C
Conditional persistence progression (0–12 h)
ndt80Δ
dmc1Δ ndt80Δ
DMC1/dmc1Δ ndt80Δ spo11-Y135F
dmc1Δ ndt80Δ spo11-Y135F
Cells with ≥1 event after time t (%)
(normalized among event-positive cells)
Time (hours)
D
Late-persistence progression (0–12 h) by genotype
ndt80Δ
dmc1Δ ndt80Δ
DMC1/dmc1Δ ndt80Δ spo11-Y135F
dmc1Δ ndt80Δ spo11-Y135F
Cells with ≥1 event after threshold (%)
Threshold time (hours)

Figure S3. Alternative summary views of nucleolar-splitting timing profiles.
(**A**) Cumulative incidence of cells with ≥1 event over the 0–12 h time course for each genotype (fraction of cells that have experienced at least one event by time t). (**B**) Per-bin distribution of events in 0.5 h intervals, expressed as the percentage of each genotype total, highlighting how events are temporally redistributed rather than simply delayed. (**C**) Conditional persistence curves ("survival" plots) showing, among event-positive cells, the fraction that still display ≥1 event after time t. (**D**) Late-persistence progression: for each genotype and threshold time, fraction of cells with ≥1 event at or beyond that threshold. Together, these complementary representations illustrate that nucleolar splitting concentrates early and wanes in *ndt80Δ*, while it remains broadly distributed into mid–late time windows in *dmc1Δ* and *spo11-y135y* backgrounds.
